# Comparative in-vitro degradation of hyaluronic acids exposed to different hyaluronidase enzymes

**DOI:** 10.1016/j.jobcr.2025.01.001

**Published:** 2025-01-13

**Authors:** Marcelo Germani, Márcia V.G.B. de Queiroz, Mariana Yuri de França Shimizu, Thiago Gomes Teixeira, Victor Rogerio, Gabriela Giro, Victor R.M. Munoz-Lora

**Affiliations:** a Department of Periodontology and Implantology, University of Guarulhos, Sao Paulo, Brazil; b Private Office, São Paulo, Brazil; c Let's HOF Academy, São Paulo, Brazil

**Keywords:** Hyaluronic acid, Hyaluronidase, Degradation, Fillers

## Abstract

**Objectives:**

This study aimed to compare the in-vitro behavior of four Hyaluronic acid (HA) gels when exposed to two different hyaluronidase (HSE) formulations.

**Methods:**

Four commercially available HA were used: Lyft (Lt; Restylane, Galderma, Sweden), Voluma (Vol; Allergan, AbbVie, USA), UltraDeep (UDe; Rennova, Innovapharma, Brazil), and Subskin (Skn; Perfectha, Sinclair, France). The gels were divided into two groups, with seven aliquots (0.1 mL per aliquot) for each group deposited on a glass plate. A millimeter ruler was positioned behind the plate to measure the gel height. Each aliquot received 100 UTR of one of two HSE formulations. After 2 min, each aliquot was mixed using a 22G needle and left to sit for additional 2 min.

**Results:**

Partial and final degradation of the products were obtained through photographic analysis. The final percentage of degradation exhibited significant differences among HA gels (P < 0.001). The results revealed higher degradation in Lt, moderate degradation in UDe, and lower degradation in Vol and Skn. Comparisons of HSE formulations showed no significant differences among them (P = 0.881). Moreover, there was a noticeable degradation after mixing (P < 0.001).

**Conclusions:**

Within the limits of this study, it can be suggested that Lt exhibits the higher degradation among the experimented gels. Furthermore, differences among HSE formulations do not appear to significantly impact HA degradation, while the mixing movement of HSE and HA seems to influence the degradation rate. These findings may help guide clinical decisions regarding the use of hyaluronidase in managing HA filler complications or adjustments.

## Graphical abstract

Quantification of the degradation of hyaluronic acid (HA) gels by hyaluronidase enzyme**.** HA gel height was measured A) before and B) after hyaluronidase application to assess the level of degradation of different HA products.

**Figure:**
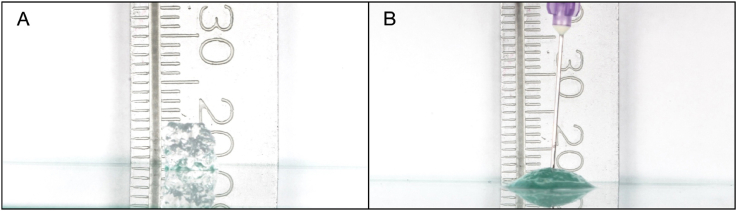

## 1 Introduction

Facial aesthetic treatments have increased significantly in recent years. According to the American Society of Plastic Surgeons, more than 3.4 million procedures were performed in 2020 in the United States. Hyaluronic acid (HA) is a high molecular weight natural polymer frequently used for facial rejuvenation.^1^ Commercially available HA undergoes modifications, gaining a unique and individual profile that varies mainly in firmness and flexibility.^2^^,^^3^

Although HA treatments are considered safe, the number of related adverse events has increased. Skin necrosis and blindness are considered the most serious events related to HA injections.^4^^,^^5^ Skin necrosis is caused by compression or obliteration of blood vessels after the application of the material. On the other hand, amaurosis (*i.e.* temporary vision loss due to lack of blood flow to the retina) is caused by a change in normal blood flow due to the injection of the material into arteries and/or veins coming from the internal carotid artery.^6^ Data reported to the FDA from 2014 to 2016 showed one case of blindness every six days and one case of necrosis every three days in patients undergoing treatments with HA.^7^

These adverse events can be resolved by the off-label, this means an indication not described in the package insert, use of the hyaluronidase enzyme (HSE), which degrades HA.^8^^,^^9^ HSE depolymerizes HA, breaking the C1 and C4 bonds of its main polymer, thus favoring its degradation. Rapid HA degradation is a key factor in achieving better outcomes from this kind of adverse events.^4^^,^^7^^,^^8^

However, despite HA gels have a similar degradation process by HSE,^10^ their differences in physicochemical properties and unique characteristics make them likely to respond different while exposed to the enzyme, even when sharing similar rheological properties and indications.^11^^,^^12^ Thus, there is still little guidance in the literature about the expected effect of HSE on these gels. Furthermore, this degradation process may also depend on HSE formulations which may vary on the extraction sources (ovine, bovine, or human recombinant), concentrations, and/or manufacturing methods,^13^ making the decision of choosing an effective HSE even more difficult in case of emergencies.

Therefore, the aim of the present study was to evaluate the level of degradation of four types of HA with similar rheological properties and clinical indications but different physicochemical characteristics, exposed to two different HSE formulations.

## 2 Materials and methods

### 2.1 HA fillers

Four commercially available HA fillers manufactured with different technologies were used in this study:

**Skn:** Subskin (Perfectha, Sinclair, France), with E-bridge technology, 20 mg of HA per mL, and about 2000 particles per mL.

**Lt:** Lyft (Restylane, Galderma, Sweden), with NASHA technology, 20 mg of HA per mL, and about 10,000 particles per mL.

**Vol:** Voluma (Allergan, AbbVie, USA), with Vycross technology, 20 mg of HA per mL.

**UDe:** UltraDeep (Rennova, Innovapharma, Brazil) Hybrid MoBi™ technology, 20 mg/mL.

### 2.2 HSE formulations

Two different commercially available and animal-source HSE formulations were used:

**Biom:** Biometil (São Bento do Sul, SC, Brazil).

**PHD:** PHD (São Caetano do Sul, SP, Brazil).

Reconstitution of both HSE was done using sterile saline solution in the proportion of 1 mL for each 2000 UTR.

### 2.3 Study design

**Fig. 1:**
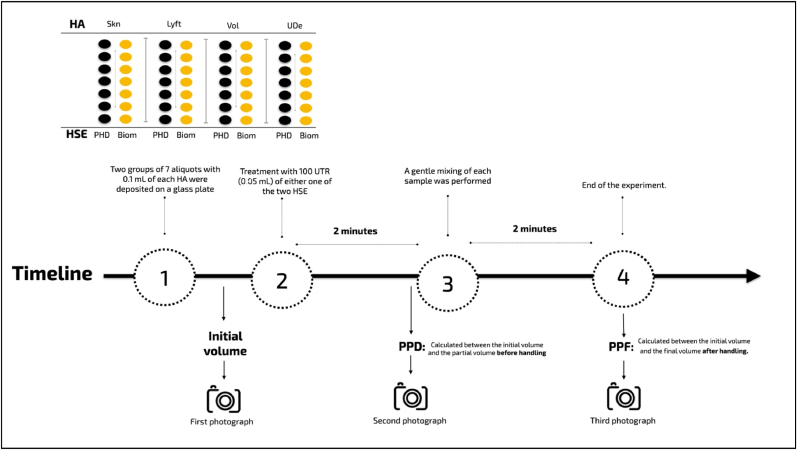
Experimental design.

**Fig. 2:**
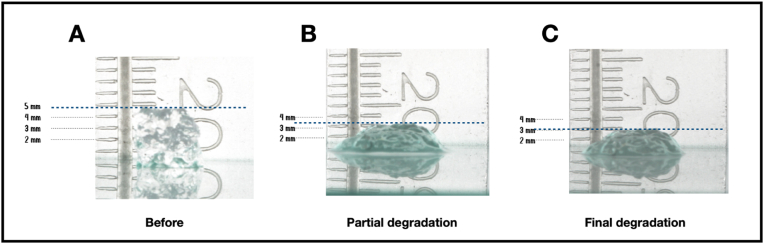
Height assessment of hyaluronic acid gels A) before, B) at partial degradation (2 min post-treatment, and C) at final degradation (2 min post-treatment and mixing).

The study design is described in Fig. 1. First, two groups of 7 aliquots containing 0.1 mL of each HA were deposited on a glass plate using a 24G x 30 mm needle (Smart GR®) positioned at a 90-degree angle in contact with the plate. Aliquots were always deposited by the same operator and using the same extrusion pressure on the syringe plunger. A millimetric ruler was positioned behind the glass plate to allow the height measurements of the gels (Fig. 2 A, B, and C).

Then, 100 UTR (0.05 mL) of either one of the two previously reconstituted HSE formulations (Biom or PHD) were dropped on the HA aliquots using a 24G x 30 mm needle (Smart GR®) and left for 2 min. The needle was never in direct contact with the product but positioned 1–2 mm away from it.

After waiting 2 min, a gentle mixing of each sample using a 24G x 30 mm needle (Smart GR®) was performed. For this, 5 gentle circular movements were made clockwise, and the aliquots were left for two more minutes before finishing the experiment.

### 2.4 Degradation assessment

The degradation assessment was conducted through standardized and reproducible photographic analysis. A digital camera (Canon T5i DSLR, Canon, USA) mounted on a tripod and equipped with a 100 mm macro lens (Canon, Japan) and a speedlight flash (Godox, São Paulo, Brazil) was used to capture images of the samples. The camera setup ensured consistent framing and lighting for all samples to minimize variability in measurements. The tripod was fixed at a height of 25 cm, maintaining a perpendicular angle (90°) to the glass plate for all photographs.

Camera settings were standardized as follows: F18 aperture, ISO 100, Neutral White Balance, autofocus, 1/125 shutter speed, and flash at 1/2 power. Photos were captured at three defined moments.

1. **Baseline (Before HSE treatment):** Photographs were taken immediately after the HA aliquots were deposited on the glass plate.
2. **After HSE Application (Partial Degradation):** Photographs were taken 2 min after the application of the HSE formulations and before any mixing.
3. **After Mixing (Final Degradation):** Photographs were taken 2 min after performing the gentle mixing movements with the needle.

All photographs included a millimeter ruler positioned vertically behind the glass plate as a reference for height measurements. Adobe Lightroom software (Adobe, California, USA) was used to adjust brightness only if necessary. The adjusted photos were then imported into Keynote (Apple, California, USA), where the images were analyzed.

The gel height was measured digitally by aligning a calibrated ruler in the software to the reference ruler visible in the images. Measurements were recorded for all aliquots at the three time points mentioned above. For consistency, all measurements were performed by the same operator, who was blinded to the HSE formulation used in each sample.

To quantify degradation, two percentages of degradation (PD) were calculated using the following formulas.

1. **Percentage of Degradation - Partial (PDP):** Calculated as the difference between the initial height (Hi) and the height before mixing (Hb), divided by the initial height:

PDP (%) = [(Hi - Hb) / Hi] × 100

2. **Percentage of Degradation - Final (PDF):** Calculated as the difference between the initial height (Hi) and the height after mixing (Ha), divided by the initial height:

PDF (%) = [(Hi - Ha) / Hi] × 100

Higher percentages indicate a greater level of degradation. This methodology ensured precise and reproducible measurements of gel height, allowing for accurate comparisons of partial and final degradation across HA fillers and HSE formulations.

## 3 Statistical analysis

The Shapiro-Wilk test was employed to assess data normality. A three-way ANOVA test was used to evaluate the influence of AH product, HSE formulation, and time on the PD. Tukey's Post Hoc test was applied to conduct individual comparisons between groups. All statistical analyses were conducted using Jamovi software (The Jamovi Project, version 1.6.23). The significance level was set at 5 %.

## 4 Results

**Fig. 3:**
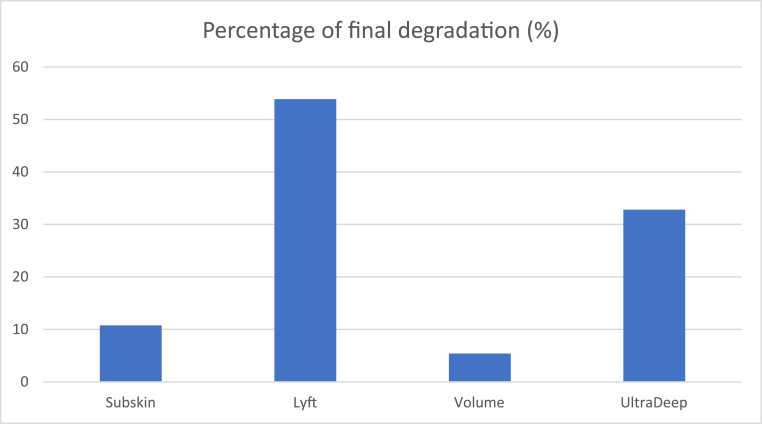
Percentage of final degradation of hyaluronic acid products disregarding the hyaluronidase formulations. The ∗symbol indicates vales significantly lower than Lyft (p < 0.05; ANOVA 3-way, Tukey's test). The + symbol indicates values significantly lower than UltraDeep (p < 0.05; Anova 3-way, Tukey's test).

In this study, we exposed four different HA fillers to two different HSE formulations. The PDF among HA products, regardless of HSE formulations, exhibited a significant difference (p < 0.001; see Fig. 3). Among these groups, Lt showed the highest degradation (p < 0.001 compared to Skn/Vol; p = 0.042 compared to UDe). UDe demonstrated significantly higher degradation than Sub and Vol (p = 0.002; 32.8 ± 22.5 vs 10.7 ± 17.5 and 5.38 ± 12.8, respectively). Importantly, there were no differences between Sub and Vol (p = 1.000).

**Fig. 4:**
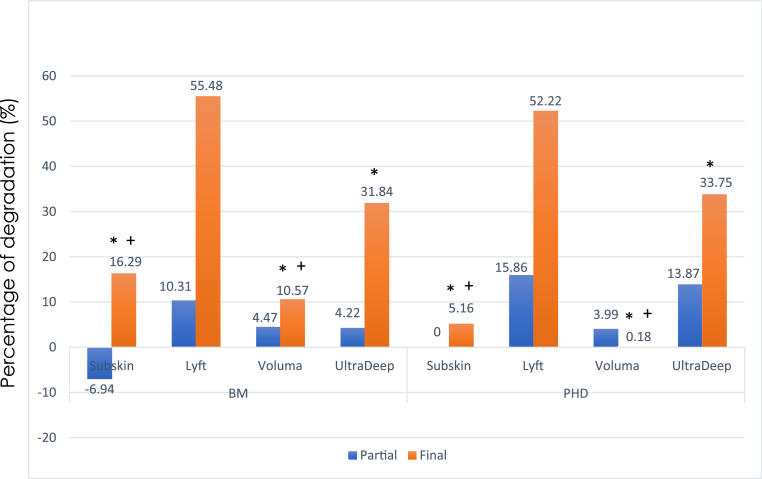
Percentage of partial and final degradation of the four hyaluronic acid groups treated with two different hyaluronidase formulations. The ∗symbol indicates values significantly lower than Lyft (p < 0.05; ANOVA 3-way, Tukey's test). The + symbol indicates values significantly lower than UltraDeep (p < 0.05; Anova 3-way, Tukey's test).

Post hoc comparisons revealed that Lt-Biom had a significantly higher PDF compared to all Vol and Skn groups (p < 0.001), with no significant differences observed with Lt-PHD and UDe groups (p > 0.05). Conversely, Vol-PHD exhibited significantly lower PDF (p < 0.05; 0.2 ± 8.8) than all other groups, except for Sub-PHD (p = 0.285; 5.2 ± 5.1). Notably, Vol-PHD was the only group where the PDP was higher than PDF (0.2 ± 8.8 vs 4.0 ± 5.7), as shown in Fig. 4.

The type of HSE formulation did not show any influence on the PDF of HA (p = 0.881). However, there was a noticeable difference among assessment times (PDF vs PDP), indicating higher degradation of HA after manipulation (p < 0.001; see Fig. 4), except for Vol-PHD (p = 1.000). Interestingly, the only product with a significant degradation difference between PDF and PDP, regardless of HSE formulation, was Lyft (p < 0.001).

## 5 Discussion

The primary objective of this study was to assess and compare the degradation of four HA gels exposed to two commercially available HSE formulations. The choice of these gels was based on their similar indication of use due to comparable rheological properties, including firmness. However, they exhibited differences in particle size, crosslinking degree, and other physicochemical characteristics. On the other hand, both HSE formulations used in this experiment are derived from animal sources and are produced by well-known manufacturers in Brazil (Biometil, São Bento do Sul, Brazil, and PHD, São Caetano do Sul, Brazil).

Our results revealed significantly higher degradation of Lt, moderate degradation of UDe, and lower degradation of Vol and Skn. It is important to note that complete gel degradation was not achieved in this study, which may be attributed to the use of a single dose of HSE and the short duration of enzymatic exposure (2 min per step). These experimental conditions were designed to simulate partial degradation under controlled in-vitro settings rather than achieve total dissolution. Furthermore, no significant differences were observed among HSE formulations, and mixing the products appeared to enhance and accelerate the degradation of HA gels by HSE.

Our findings align with previous research indicating Lyft as a gel that readily dissolves. In 2018, Ferraz et al. described a rapid degradation of NASHA-technology gels, including Lyft, using an in-vivo model.^11^ Also, a correlation between the degradation level and the technology of HA gels was demonstrated, with OBT and NASHA technologies displaying similar results. In a different experiment,^12^ Restylane Lyft was also identified as an easily dissolvable gel among 12 different products, irrespective of the HSE dose used. In contrast, Juvéderm Voluma, exhibited minimal dissolution. Casabona et al.^14^ also compared five different HA gels and found Juvéderm Voluma to be less susceptible to dissolution, a finding that can be corroborated with our results.

It's well-established that HA reversibility vary based on the physicochemical characteristics of the products, and no single variable such as crosslinking degree, particle size, or concentration can predict a gel response to HSE.^11^^,^^14^ These characteristics can also influence the aesthetic outcomes, tissue behavior, and product longevity. Importantly, all products examined in our research have been studied in the literature regarding their physicochemical and rheological properties.^2^^,^^15^, ^16^, ^17^

Two critical characteristics to consider are the degree of modification (i.e. crosslinking) and HA concentration. Apparently, less crosslinked HA tend to be less resistant to HSE degradation,^12^ as confirmed in our research. Lt has one of the lowest modification degrees in the market (1 %), potentially explaining the higher degradation rate.^18^ Crosslinking details for the other products are not provided by the manufacturers. On the other side, all HA products in our study had the same HA-concentration (20 mg/mL), so no correlation between this characteristic and the level of degradation can be established.

The accessibility of HSE to HA gels is another crucial factor in HA degradation.^11^ Our study demonstrated significantly greater gel degradation after mixing with a needle. Mixing may allow a direct access of the injected HSE to the HA gel, resulting in a higher superficial contact and higher degradation. This outcome may support the idea of gentle massaging after HSE injection in cases of vascular occlusion, to aid in the breakdown of crosslinked HA.^8^ However, it's important to clarify that this paper does not intend to provide clinical guidance after complications.

An outstanding aspect of this study lies in the quantitative methodology of the in-vitro analysis. In contrast to other studies that subjectively assessed the reduction in the size and/or height of the papule formed after enzyme application, our approach minimizes potential misinterpretations due to different observations. In addition, comparing products with similar rheological properties but different physicochemical characteristics help us to understand the possible factors that may influence HA degradation rates. Among the limitations of this study, it is worth mentioning that only a single dose of HSE was used. Different HSE concentrations may lead to distinct results. Furthermore, given the limited data available on HA gel reversibility, our results contribute to a deeper understanding of the variations in HA degradation levels.

## 6 Conclusion

The findings from this study indicate varying degrees of degradation in response to HSE, with Lt showing a higher degradation degree among other HA gels. These results suggest that HSE formulations may not significantly influence the degradation profile of HA gels, but the application of physical movement appears to enhance its effectiveness degrading HA. These results may reinforce the need of a local massage if total degradation of a HA gels is needed in a clinical situation. However, given the extensive range of commercially available HA products and the different physicochemical characteristics of them, further comparative experiments are warranted to provide a more comprehensive understanding of their behavior.

## Patient consent

Do not apply for in-vitro studies.

## Ethical clearance

This is in-vitro research that need no ethical committee acceptance.

## Source of funding

This research received no funding.

## Declaration of competing interest

The authors declare that they have no known competing financial interests or personal relationships that could have appeared to influence the work reported in this paper.
